# Radiosynthesis and quality control testing of two ^18^F-labeled radiopharmaceuticals for α-synuclein imaging in clinical investigations

**DOI:** 10.1186/s41181-026-00480-w

**Published:** 2026-07-23

**Authors:** Hiroki Hashimoto, Takayuki Ohkubo, Kazunori Kawamura, Tomoya Fujishiro, Daisuke Arashi, Tatsuto Sato, Takeru Seki, Masatoshi Muto, Kaito Tsukagoe, Masanao Ogawa, Yusuke Kurihara, Nobuki Nengaki, Wakana Mori, Masayuki Fujinaga, Makoto Higuchi, Ming-Rong Zhang

**Affiliations:** 1 Department of Advanced Nuclear Medicine Sciences, Institute for Quantum Medical Science, National Institutes for Quantum Science and Technology, 4-9-1 Anagawa, Inage-ku, Chiba, 263-8555 Japan; 2 SHI Accelerator Service Ltd., 7-1-1 Nishigotanda, Shinagawa-ku, Tokyo, 141-0032 Japan; 3 Tokyo Nuclear Services Co., Ltd., 1-3-5 Taito, Taito-ku, Tokyo, 110-0016 Japan; 4 Advanced Neuroimaging Center, Institute for Quantum Medical Science, National Institutes for Quantum Science and Technology, 4-9-1 Anagawa, Inage-ku, Chiba, 263-8555 Japan; 5 Neuroetiology and Diagnostic Science, Osaka Metropolitan University Graduate School of Medicine, 1-4-3 Asahi-Machi, Abeno-ku, Osaka, 545-8585 Japan

**Keywords:** ^18^F, Radiopharmaceutical, SPAL-T-06, C05-05, α-Synuclein, Radiosynthesis, Positron emission tomography

## Abstract

**Background:**

^18^F-Labeled (*E*)-1-fluoro-3-((2-(4-(5-(methylamino)pyrazin-2-yl)but-1-en-3-yn-1-yl)benzo[*d*]thiazol-6-yl)oxy)propan-2-ol ([^18^F]SPAL-T-06) and (*E*)-1-fluoro-3-((2-(4-(6-(methylamino)pyridine-3-yl)but-1-en-3-yn-1-yl)benzo[d]thiazol-6-yl)oxy)propan-2-ol ([^18^F]C05-05) are positron emission tomography (PET) imaging agents for α-synuclein pathologies. Recently, we demonstrated the utility of PET imaging with [^18^F]SPAL-T-06 for α-synuclein pathologies in patients with multiple system atrophy and with [^18^F]C05-05 for α-synuclein pathologies in Parkinson’s disease and related disorders. In this study, to establish [^18^F]SPAL-T-06 and [^18^F]C05-05 as PET radiopharmaceuticals for clinical investigations, we developed routine radiosynthetic procedures using an ^18^F-labeling synthesizer and performed quality control (QC) testing of these two PET radiopharmaceuticals.

**Results:**

[^18^F]SPAL-T-06 and [^18^F]C05-05 were synthesized by direct ^18^F-fluorination of their respective tetrahydropyranyl-protected tosylated precursors, followed by deprotection, using an ^18^F-labeling synthesizer. The non-decay-corrected radiochemical yields of [^18^F]SPAL-T-06 and [^18^F]C05-05 from ^18^F^−^ at the end of synthesis (EOS) were 11 ± 4.6% (*n* = 19) and 20 ± 6.8% (*n* = 37), respectively. The radiochemical purities of both PET radiopharmaceuticals exceeded 99%. The molar activities of each PET radiopharmaceutical at EOS exceeded 68 GBq/µmol, and the total synthesis times were within 100 min after irradiation. In addition, [^18^F]SPAL-T-06 and [^18^F]C05-05 were efficiently separated from byproducts using a semi-preparative high-performance liquid chromatography system. All QC results for the two PET radiopharmaceuticals complied with in-house QC and quality assurance specifications. The stabilities of the two PET radiopharmaceuticals were evaluated under standard fluorescent light in a general laboratory environment and under UV-cutoff light in a natural light-shielded environment. After 10 min of exposure to the standard fluorescent light, the radiochemical purity of each tracer decreased to approximately 86%. In contrast, the radiochemical purity of each tracer exceeded 99% after 60 min under UV-cutoff light in a natural light-shielded environment.

**Conclusions:**

We successfully prepared two PET radiopharmaceuticals, [^18^F]SPAL-T-06 and [^18^F]C05-05, for clinical investigation. The radiosynthesis and QC findings provide a practical basis for establishing standardized production procedures and facilitating technology transfer to other PET centers. In particularly, all post-deprotection radiosynthesis steps and QC testing for [^18^F]SPAL-T-06 and [^18^F]C05-05 should be performed under UV-cutoff light in a natural light-shielded environment to prevent photoisomerization.

**Supplementary Information:**

The online version contains supplementary material available at https://doi.org/10.1186/s41181-026-00480-w.

## Background

**Fig. 1:**
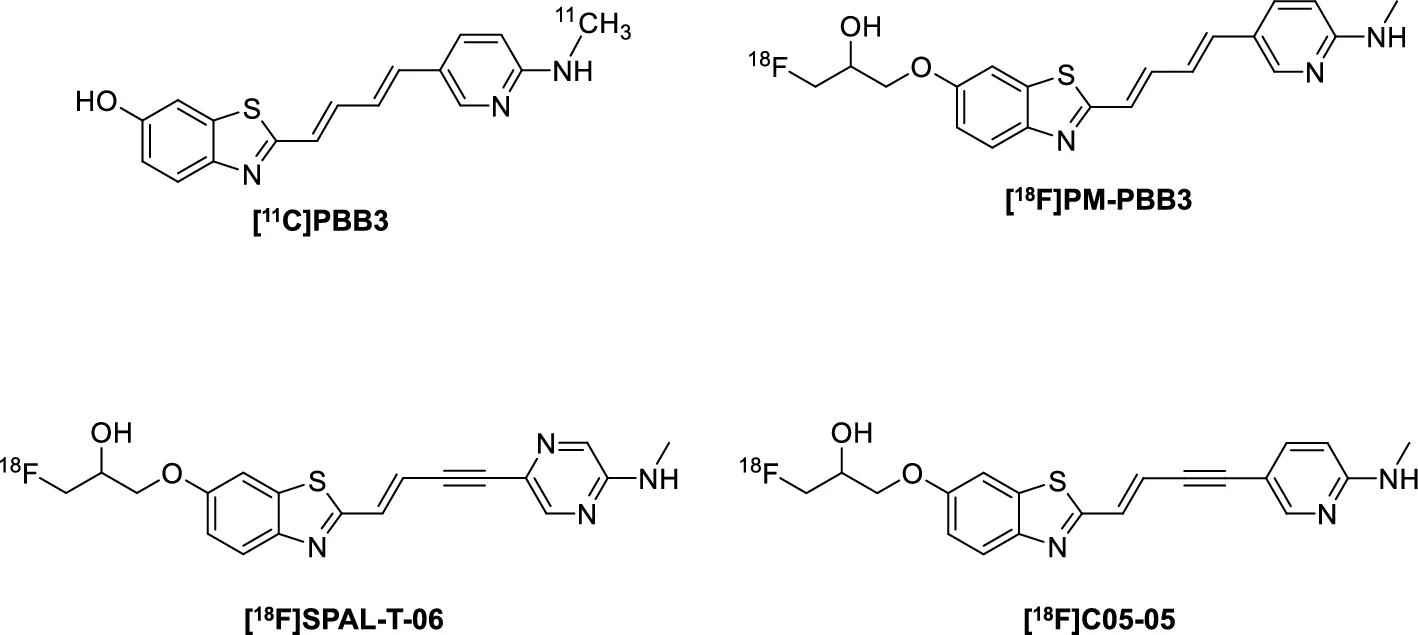
Chemical structures of [^11^C]PBB3, [^18^F]PM-PBB3, [^18^F]SPAL-T-06, and [^18^F]C05-05

^18^F-Labeled (*E*)-1-fluoro-3-((2-(4-(5-(methylamino)pyrazin-2-yl)but-1-en-3-yn-1-yl)benzo[*d*]thiazol-6-yl)oxy)propan-2-ol ([^18^F]SPAL-T-06, Fig. 1) and (*E*)-1-fluoro-3-((2-(4-(6-(methylamino)pyridine-3-yl)but-1-en-3-yn-1-yl)benzo[*d*]thiazol-6-yl)oxy)propan-2-ol ([^18^F]C05-05, Fig. 1) were developed for imaging α-synuclein inclusions in the brain (Endo et al. 2024; Goto et al. 2025; Matsuoka et al. 2022). Recently, we developed the ^11^C-labeled 2-((1*E*,3*E*)-4-(6-(methylamino)pyridin-3-yl)buta-1,3-dienyl)benzo[*d*]thiazol-6-ol ([^11^C]PBB3, Fig. 1) and ^18^F-labeled 1-fluoro-3-((2-((1*E*,3*E*)-4-(6-(methylamino)pyridine-3-yl)buta-1,3-dien-1-yl)benzo[*d*]thiazol-6-yl)oxy)propan-2-ol ([^18^F]PM-PBB3, [^18^F]APN-1607, or [^18^F]florzolotau, Fig. 1) as clinically used positron emission tomography (PET) radiopharmaceuticals for imaging tau pathology in the human brain (Hashimoto et al. 2014; Kawamura et al. 2021; Maruyama et al. 2013; Tagai et al. 2021). Notably, substitution of the (2*E*,4*E*)-hexa-2,4-dien linker in the chemical structure of PBB3 series compounds, including PM-PBB3, with (*E*)-hex-2-en-4-yne resulted in a marked increase in ligand binding to α-synuclein relative to tau and Aβ fibrils, leading to the generation of C05-05 or SPAL-T-06 (Endo et al. 2024; Higuchi et al. 2025; Matsuoka et al. 2022). The linker substitution may produce differences in the backbone twist angle between the PBB3 and C05 series of compounds in the minimum energy state, which may affect the conformational fitness of the compound for the binding surface of filaments with a unique distortion angle (Higuchi et al. 2025; Miranda-Azpiazu et al. 2020). The IC_50_ values of C05-05 (1.5 nM) and SPAL-T-06 (1.6 nM) for homologous binding blockade were approximately 40- and 18-fold lower than those of PBB3 and PM-PBB3, respectively, implying that the linker angle plays a critical role in ligand affinity for pathological fibrils (Endo et al. 2024; Matsuoka et al. 2022). Based on these insights, it is necessary to assess the in vitro interactions of C05-05 and SPAL-T-06 with aggregates in various α-synucleinopathies, which may provide useful information for predicting the in vivo performance of the probes in clinical PET imaging of related disorders (Endo et al. 2024; Matsuoka et al. 2022). More recently, we demonstrated the utility of PET imaging using [^18^F]SPAL-T-06 for α-synuclein pathologies in patients with multiple system atrophy, achieving high contrast and enabling image interpretation at the individual level for diagnostic purposes (Matsuoka et al. 2022). Moreover, we demonstrated the utility of PET imaging using [^18^F]C05-05 for α-synuclein pathologies in clinical investigations (Endo et al. 2024). In addition, radiation dosimetry of [^18^F]SPAL-T-06 and [^18^F]C05-05 in humans has been evaluated (Goto et al. 2025).

In this study, to establish [^18^F]SPAL-T-06 and [^18^F]C05-05 as PET radiopharmaceuticals for clinical investigations, we developed routine radiosynthetic procedures using an ^18^F-labeling synthesizer and performed quality control (QC) testing.

## Methods

### General

SPAL-T-06 (Fig. 1) and its precursor (SPAL-T-30) for ^18^F labeling were provided by Takeda Pharmaceutical Company Limited (Osaka, Japan). C05-05 (Fig. 1) and its precursor for ^18^F labeling were purchased from the Nard Institute (Kobe, Japan). Polysorbate 80 (Tween 80) was purchased from MP Biomedicals (Irvine, CA, USA). Distilled water for injection and saline were purchased from Otsuka Pharmaceutical Factory (Naruto, Japan). Ascorbic acid (500 mg/2 mL) was purchased from Fuso Pharmaceutical Industries (Osaka, Japan). All other reagents and organic solvents were purchased from Merck (Darmstadt, Germany), FUJIFILM Wako Pure Chemical (Osaka, Japan), and Nacalai Tesque (Kyoto, Japan), and were used without further purification.

The two PET radiopharmaceuticals were produced using an automated multipurpose radiosynthesis system equipped with an ^18^F-labeling synthesizer developed at our institute (Zhang et al. 2002).

Semi-preparative high-performance liquid chromatography (HPLC) was performed using a radio-HPLC system, in which a Jasco HPLC system (PU-2080 pump and UV-2070 detector; Jasco, Tokyo, Japan) was combined with an NaI(Tl) scintillation detector system (OHYO KOKEN KOGYO, Tokyo, Japan). The semi-preparative HPLC conditions were as follows: an Inertsil ODS-HL column (5 μm, 10 mm internal diameter [i.d.] × 250 mm length; GL Sciences, Tokyo, Japan); a mixture of acetonitrile and 50 mmol/L ammonium acetate solution (44:56 [v/v] for [^18^F]SPAL-T-06 or 50:50 [v/v] for [^18^F]C05-05) as the mobile phase; a flow rate of 5.0 mL/min; and UV detection at 390 nm.

Analytical HPLC was performed using a radio-HPLC system, in which a Waters HPLC system (515 pump and 2489 UV detector; Waters, Milford, MA, USA) was combined with an NaI(Tl) scintillation detector system (Gabi Star PET; Raytest, Straubenhardt, Germany). The analytical HPLC conditions were as follows: an ACE Excel 3 C18-Amide column (3 μm, 4.6 mm i.d. × 100 mm; Advanced Chromatography Technologies, Aberdeen, UK); a mixture of 90% acetonitrile and 100 mmol/L ammonium phosphate buffer (pH 2.0) containing 5 mmol/L sodium 1-octanesulfonate (42:58 [v/v] for [^18^F]SPAL-T-06 or 35:65 [v/v] for [^18^F]C05-05) as the mobile phase; a flow rate of 1.0 mL/min; UV detection at 390 nm; and an injection volume of 5 μL.

Gas chromatography (GC) was performed using an Agilent 7890 Series GC system (G3440A; Agilent Technologies, Santa Clara, CA, USA). The GC conditions were as follows: 0.5 μL injection volume; a DB-WAX column (0.50 μm, 0.53 mm i.d. × 30 m; Agilent Technologies); the column temperature was initially set at 40 °C (0–8 min), then increased to 120 °C (8–28 min), and finally maintained at 120 °C (28–33 min); nitrogen (99.99995%, 50 mL/min) was used as the makeup gas; helium (99.99995%, 4 mL/min) was used as the carrier gas; and detection was performed using a flame ionization detector with hydrogen (99.9999%) and compressed air as the combustion gases. Mass was measured using an electronic balance (BL220H; Shimadzu, Kyoto, Japan). Radioactivity was determined using a dose calibrator (CRC-25R; CAPINTEC, Florham Park, NJ, USA). Radionuclidic identity and purity were measured by gamma spectroscopy using a Ge semiconductor detector system (detector: GEM-10175-P; ORTEC, Oak Ridge, TN, USA; multichannel analyzer: MCA-7a; SEIKO EG&G, Tokyo, Japan). pH was measured using a pH meter (F-72; HORIBA, Kyoto, Japan) equipped with a probe (9618S-10D; HORIBA). Bacterial endotoxin tests were performed using a toxinometer (ET-6000; FUJIFILM Wako Pure Chemical Industries, Osaka, Japan) and a Limulus ES-II Test Wako (FUJIFILM Wako Pure Chemical Industries).

### Radiosyntheses of [^18^F]SPAL-T-06 and [^18^F]C05-05

**Fig. 2:**
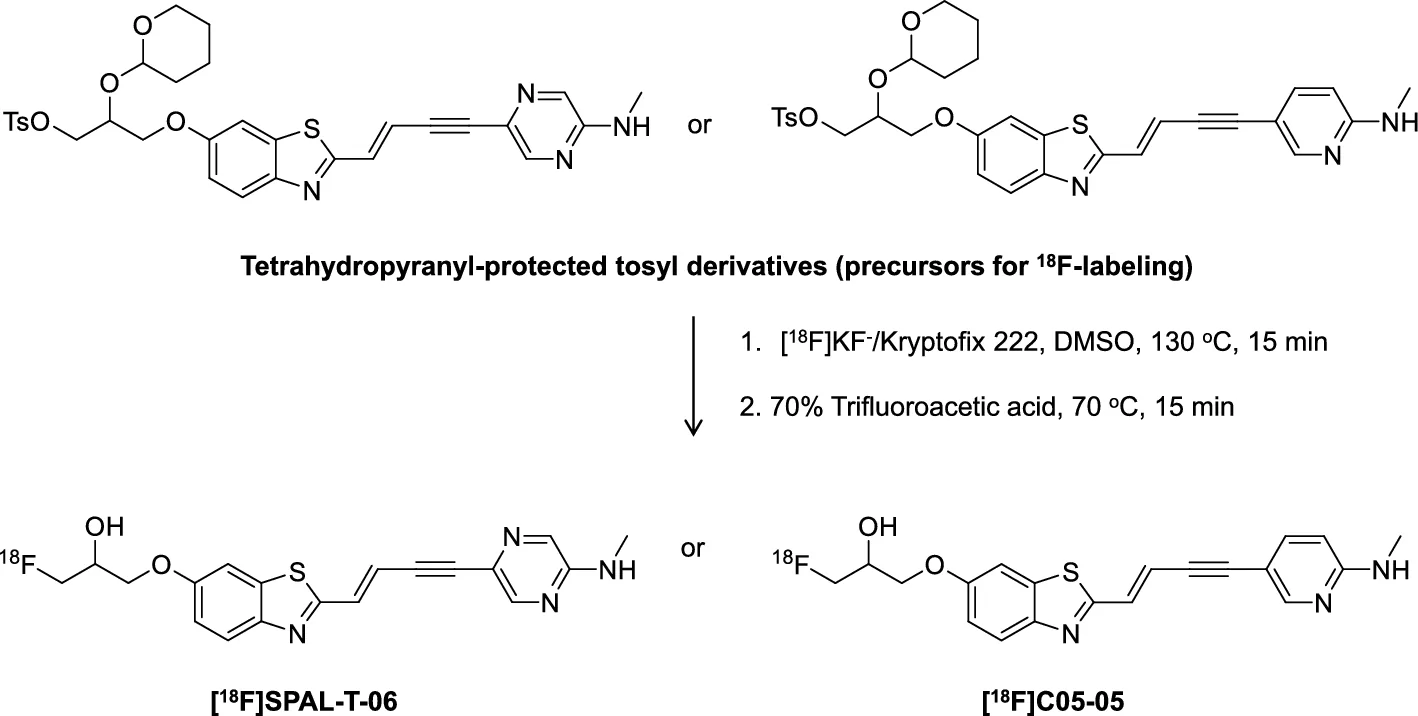
Radiosyntheses of [^18^F]SPAL-T-06 and [^18^F]C05-05

[^18^F]SPAL-T-06 and [^18^F]C05-05 were synthesized by direct ^18^F fluorination of their respective tetrahydropyranyl (THP)-protected tosylated precursors, followed by deprotection (Fig. 2), using an ^18^F-labeling synthesizer (Zhang et al. 2002). The radiosyntheses of [^18^F]SPAL-T-06 and [^18^F]C05-05 are shown in Fig. 2 and described in detail below.

**Fig. 3:**
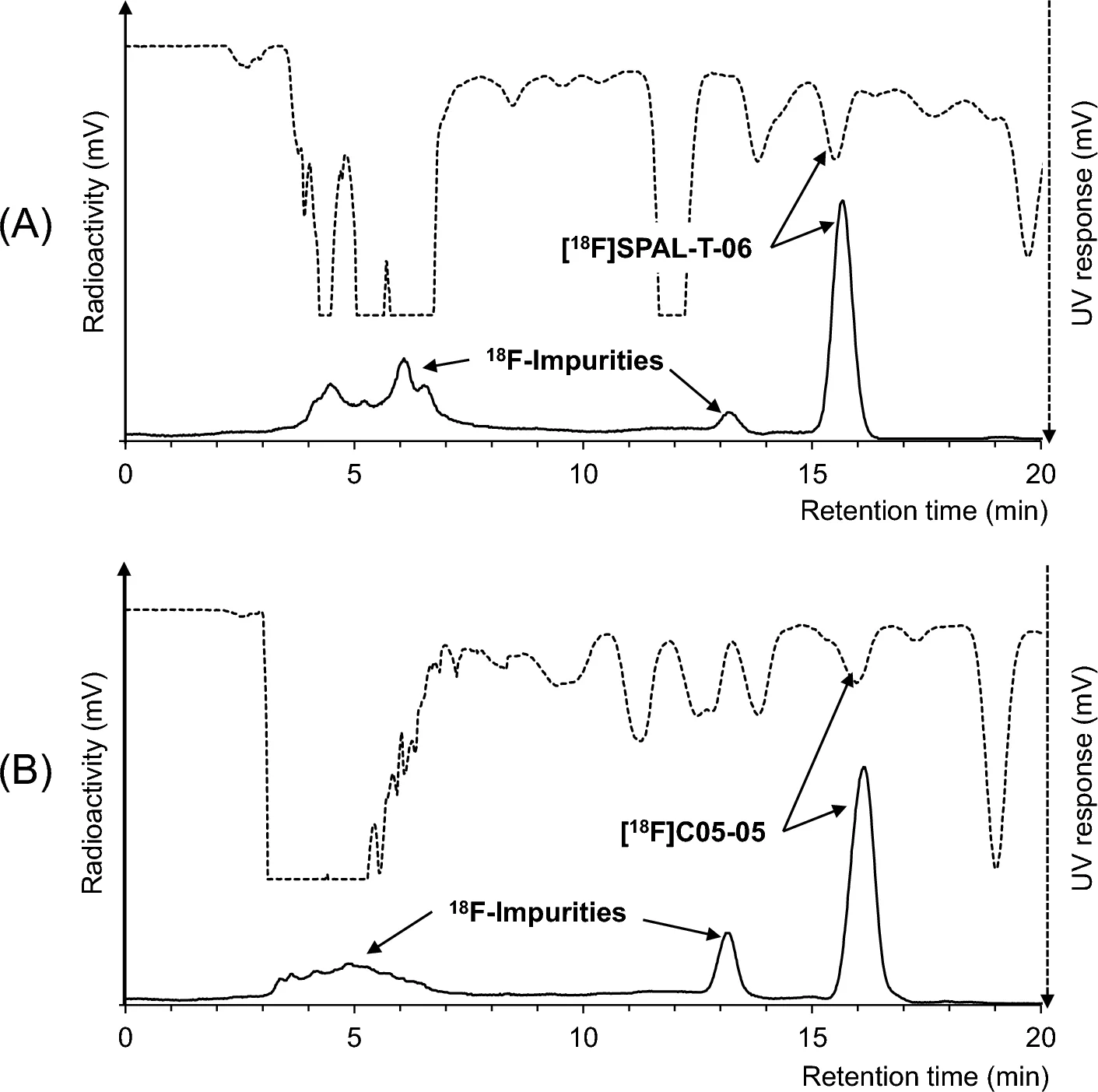
Typical semi-preparative HPLC chromatograms of [^18^F]SPAL-T-06 (**A**) and [^18^F]C05-05 (**B**) obtained using radio-HPLC. Semi-preparative HPLC conditions were as follows: an Inertsil ODS-HL column (5 μm, 10 mm i.d. × 250 mm), acetonitrile/50 mmol/L ammonium acetate solution (44:56 [v/v] for [^18^F]SPAL-T-06 or 50:50 [v/v] for [^18^F]C05-05) as the mobile phase, a flow rate of 5.0 mL/min, and UV detection at 390 nm

Fluorine-18, as [^18^F]F^−^, was produced in a cyclotron (BC2010; Japan Steel Works, Tokyo, Japan; Sumitomo Heavy Industries, Ltd., Tokyo, Japan) via the ^18^O(p,n)^18^F reaction in > 98 atomic % ^18^O-enriched water (Taiyo Nippon Sanso, Tokyo, Japan) using 18 MeV protons. [^18^F]F^−^ was separated from H_2_^18^O by solid-phase extraction using a Sep-Pak Accell Plus QMA Plus Light cartridge (Waters). Before extraction, the cartridge was preconditioned sequentially with 10 mL of ethanol, 10 mL of water for injection, 10 mL of 0.5 mol/L potassium carbonate solution, and 10 mL of water for injection. [^18^F]F^−^ was eluted from the cartridge with a mixture of 10 mmol/L potassium carbonate solution (0.41 mg/0.25 mL) and a solution of Kryptofix 222 in acetonitrile (9.4 mg/0.25 mL) and transferred to a reaction vessel. The aqueous [^18^F]F^−^ solution was gradually dried at 80–130 °C for 15–30 min to completely remove water and acetonitrile. After removal of the solvents, the THP-protected tosylated precursor (2.0 mg) in anhydrous dimethyl sulfoxide (DMSO, 0.3 mL) was added to the reaction vessel and the mixture was heated at 130 °C for 15 min. Following ^18^F fluorination, 70% aqueous trifluoroacetic acid (TFA) solution (0.6 mL) was added to the reaction vessel, and the mixture was heated at 70 °C for 15 min. After deprotection, a mixture of 4 mol/L sodium acetate (1.5 mL) and DMSO (0.5 mL) was added to the reaction vessel. After deprotection, all radiosynthesis processes were performed under UV-cutoff light (< 500 nm cutoff wavelength; yellow light; ECOHiLUX HES-YF, Iris Ohyama, Sendai, Japan, or Brinis MTAXS20YM, Tnet, Tokyo, Japan) in a natural light-shielded environment to prevent photoisomerization. The reaction mixture was then transferred to an injector for semi-preparative HPLC as described above. The retention times of [^18^F]SPAL-T-06 and [^18^F]C05-05 were approximately 15.6 and 16.1 min, respectively (Fig. 3). The HPLC fractions containing [^18^F]SPAL-T-06 and [^18^F]C05-05 were collected in a flask; polysorbate 80 (75 μL) in ethanol (0.3 mL) and ascorbic acid (25 mg/0.1 mL) had been added to the flask before radiosynthesis. The solution was subsequently evaporated to dryness under reduced pressure at 300 °C, and the residue was dissolved in physiological saline (10–17 mL). The resulting solution was transferred to a sterile isolator (JFE Engineering, Tokyo, Japan) as previously described (Waki et al. 2016). Briefly, in the sterile isolator, the resulting solution was passed through a Millex-GV filter (Merck) to obtain [^18^F]SPAL-T-06 or [^18^F]C05-05, which was automatically dispensed and transferred to a sterile vial under UV-cutoff light in a natural light-shielded environment. Filter integrity testing of the Millex-GV filter was automatically performed after dispensing each solution using the bubble point test (criteria value, over 291 kPa) and a manometer calibrated in accordance with the Japanese Industrial Standards.

### QC testing for PET radiopharmaceuticals

All QC testing procedures were performed in accordance with the Japanese Society of Nuclear Medicine Good Manufacturing Practice guidelines (2nd Edition, February 2026) and the Japanese Pharmacopoeia (17th Edition) (Table 1). The specifications, methods, and acceptance criteria for QC are listed in Table 1. The QC methods have been described previously (Kawamura et al. 2021) and in the General section. The details of the molar activity, radiochemical identity, radiochemical purity, residual solvents, Kryptofix 222, and bacterial endotoxins are described bellows. Molar activity, radiochemical identity, and radiochemical purity were measured using radio-HPLC under UV-cutoff light in a natural light-shielded environment to prevent photoisomerization. The retention times of [^18^F]SPAL-T-06 and [^18^F]C05-05 were approximately 11.9 and 10.3 min, respectively. Their identities were confirmed by co-injection with the corresponding authentic non-radiolabeled compounds. Molar activity was calculated by comparing the measured radioactivity with the carrier mass determined from the UV peak at 390 nm and calibration curves (0.01, 0.1, 1, and 10 μg/mL of each solution as a reference standard; Fig. S1). Residual solvents (acetonitrile, ethanol and DMSO) were determined using quantitative analyses (calibration curves using 10, 100, and 1,000 μg/mL of each solution as a reference standard; Fig. S2). The amount of Kryptofix 222 was determined using thin-layer chromatography (plate: TLC Silica gel 60 F254, Merck; mobile phase: methanol/25% ammonia solution, 9:1 [v/v]; detection: iodine stain; and injection volume: 2 μL) and the limit test (40 μg/mL of Kryptofix 222 solution as a reference standard).

**Table 1:** Specifications, methods, and acceptance criteria for quality control of the [^18^F]SPAL-T-06 and [^18^F]C05-05 injections

Specification	Methods	Acceptance criteria
Volume	Measured using an electronic balance	10–18 mL
Radioactivity	Measured using a dose calibrator	0.185–10 GBq
Molar activity	Radio-HPLC	≥ 37 GBq/µmol
Half-life	Measured using a dose calibrator	105–115 min
Appearance	Visual inspection	A clear, colorless liquid with no suspended particles
Bacterial endotoxin	Measured using a toxinometer	< 150 international units per maximum dose
Sterility	Post-release (Japanese Pharmacopoeia)	No bacterial growth
pH	Measured using a pH meter	5.0–8.0
Radionuclidic identity	Gamma spectrometry	A gamma emission corresponding to 511 keV
Radiochemical identity	Radio-HPLC	Percent difference in retention time between radioactivity peak and reference UV peak should be within ± 15%
Radionuclidic purity	Gamma spectrometry	≥ 99.9% of the total observed gamma emissions correspond to 511 keV and 1022 keV
Radiochemical purity	Radio-HPLC	≥ 95%
Kryptofix 222	Thin-layer chromatography	< 40 µg/mL
Residual solvents Ethanol Acetonitrile Dimethyl sulfoxide	Gas chromatography with flame ionization detection	< 5000 µg/mL* < 410 µg/mL* < 5000 µg/mL*

^*^Estimated values assuming a maximum injection volume of 10 mL

### Stabilities of [^18^F]SPAL-T-06 and [^18^F]C05-05 under standard fluorescent light or UV cut-off light

The stabilities of [^18^F]SPAL-T-06 and [^18^F]C05-05 under standard fluorescent light (FHF32EX-N-H, 32W, Panasonic Electric Works Co., Tokyo, Japan) in a general laboratory environment and UV cut-off light in a natural-light shielded environment were evaluated by determining the radiochemical purity of the injection product using radio-HPLC, as described in the General section. Under standard fluorescent light in a general laboratory environment, approximately 1 mL of each injection was transferred into a capped clear glass vial and positioned 70 cm below the fluorescent light (range: 760–930 lx), and maintained for 10, 30, and 60 min at 15–25 °C. Under the UV-cutoff light in a natural light-shielded environment, approximately 1 mL of each injection was transferred into a capped clear glass vial and positioned 50 cm below UV-cutoff light (approximately 670 lx), and maintained for 60 min at 15–25 °C. Furthermore, the stabilities of [^18^F]SPAL-T-06 and [^18^F]C05-05 were evaluated for 180 min after radiosynthesis in a natural light-shielded environment. A sample of each injection (approximately 1 mL) was transferred into a capped clear glass vial within a lead-shielded container covering the side and bottom of the vial in a natural light-shielded environment (non-fluorescent/UV-cutoff light) and maintained for 180 min at 15–25 °C.

### Statistical analysis

Quantitative data are expressed as the mean ± standard deviation. Differences among the three groups were analyzed using the Kruskal–Wallis one-way analysis of variance followed by Dunn’s test. Data were analyzed using OriginPro software (version 2026; OriginLab Corporation, Northampton, MA, USA). Differences were considered statistically significant at *p* < 0.05.

## Results

### Radiosyntheses of [^18^F]SPAL-T-06 and [^18^F]C05-05

Initially, we synthesized [^18^F]SPAL-T-06 under the same reaction conditions as those used for the radiosynthesis of [^18^F]PM-PBB3 (Kawamura et al. 2021). Unexpectedly, the non-decay-corrected radiochemical yield (RCY [n.d.c]) of [^18^F]SPAL-T-06 from [^18^F]F^−^ at the end of synthesis (EOS) was only 4.7% (Table 2), which was lower than that of [^18^F]PM-PBB3 (25%**;** Table 3; Kawamura et al. 2021). To achieve efficient radiosynthesis of [^18^F]SPAL-T-06, we selected TFA as the deprotection reagent instead of hydrochloric acid, as previously described (Kawamura et al. 2021). When 50% TFA was used for deprotection, the RCY (n.d.c) at EOS increased to 7.9%. We subsequently decreased the reaction temperature for removal of the THP group from 90 to 70 °C because the boiling point of TFA is 72 °C. As a result, the RCY (n.d.c) of [^18^F]SPAL-T-06 at EOS increased to 9.9%. In addition, increasing the TFA concentration from 50 to 70% further increased the RCY (n.d.c) at EOS to 11.4% (Table 2).

**Table 2:** Examination of deprotection conditions for the radiosynthesis of [^18^F]SPAL-T-06

Deprotection		Diluent (mL)	Radiochemical yield (%)*
Reagent	Reaction conditions		
4 mol/L HCl	90 °C, 10 min	2 mol/L sodium acetate (1.0)	4.7
50% TFA	90 °C, 10 min	4 mol/L sodium acetate (1.5) + DMSO (0.5)	7.9
50% TFA	70 °C, 15 min	4 mol/L sodium acetate (1.5) + DMSO (0.5)	9.9
70% TFA	70 °C, 15 min	4 mol/L sodium acetate (1.5) + DMSO (0.5)	11.4

^*^Radiochemical yield based on [^18^F]F^−^ (non-decay-corrected value at the end of synthesis); single exploratory experiment

**Table 3:** Radiosynthesis results of [^18^F]SPAL-T-06, [^18^F]C05-05, and [^18^F]PM-PBB3

	[^18^F]SPAL-T-06^a^	[^18^F]C05-05^b^	[^18^F]PM-PBB3^c^
Radiochemical yield (%)*	11 ± 4.6^#^	20 ± 6.8^#^	25 ± 6.0^#^
Radiochemical purity (%)*	99.7 ± 0.346	99.6 ± 0.485	98 ± 0.58
Molar activity (GBq/μmol)*	390 ± 230	280 ± 130	350 ± 94

^*^Non-decay-corrected values at the end of synthesis. ^a^Mean ± standard deviation (*n* = 19). ^b^Mean ± standard deviation (*n* = 37). ^c^Mean ± standard deviation (*n* = 53; Kawamura et al. 2021). ^#^*p* < 0.05 (Kruskal–Wallis one-way analysis of variance followed by Dunn’s test; comparisons of radiochemical yields between [^18^F]SPAL-T-06 and [^18^F]C05-05, [^18^F]SPAL-T-06 and [^18^F]PM-PBB3, and [^18^F]C05-05 and [^18^F]PM-PBB3)

**Fig. 4:**
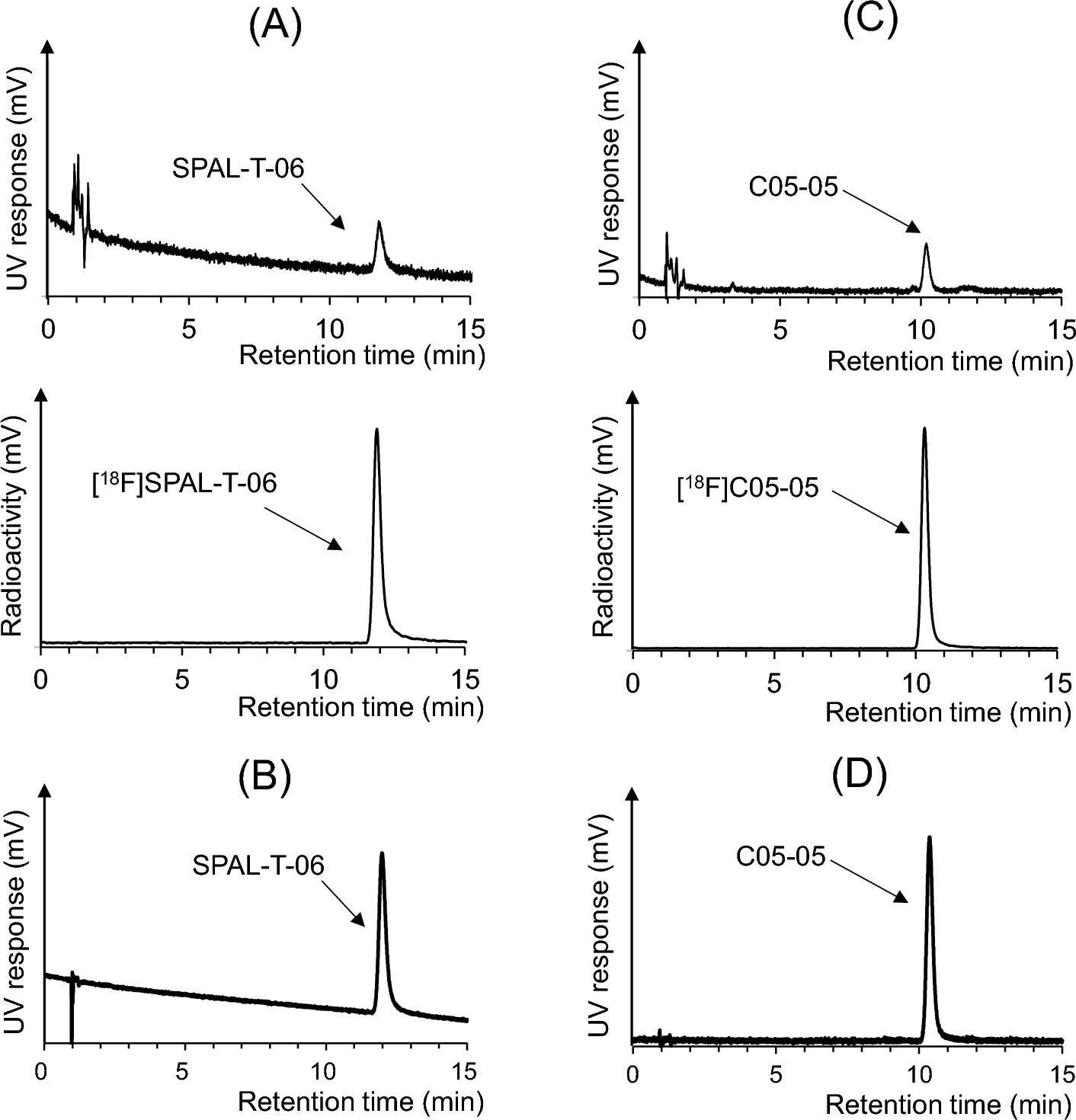
Typical analytical HPLC chromatograms of [^18^F]SPAL-T-06 (**A**), non-radiolabeled SPAL-T-06 as a reference standard (**B**), [^18^F]C05-05 (**C**), and non-radiolabeled C05-05 as a reference standard (**D**) obtained using radio-HPLC. The analytical HPLC conditions were as follows: an ACE Excel 3 C18-Amide column (3 μm, 4.6 mm i.d. × 100 mm), a mixture of 90% acetonitrile and 100 mmol/L ammonium phosphate buffer (pH 2.0) containing 5 mmol/L sodium 1-octanesulfonate (42:58 [v/v] for [^18^F]SPAL-T-06 or 35:65 [v/v] for [^18^F]C05-05) as the mobile phase, a flow rate of 1.0 mL/min, UV detection at 390 nm, and an injection volume of 5 μL. The retention times of [^18^F]SPAL-T-06 and [^18^F]C05-05 were 11.9 and 10.3 min, respectively

Under the examined deprotection conditions, we successfully prepared [^18^F]SPAL-T-06 for routine clinical applications using an ^18^F-labeling synthesizer. We also successfully prepared [^18^F]C05-05 under the same reaction conditions as those used for the radiosynthesis of [^18^F]SPAL-T-06. In addition, [^18^F]SPAL-T-06 and [^18^F]C05-05 were efficiently separated from byproducts using a semi-preparative HPLC system (Figs. 3 and S3). As shown in Table 3, the RCYs (n.d.c) of [^18^F]SPAL-T-06 and [^18^F]C05-05 from [^18^F]F^−^ at EOS were 11 ± 4.6% (*n* = 19) and 20 ± 6.8% (*n* = 37), respectively. The radiochemical purities of the two PET radiopharmaceuticals exceeded 99% (Fig. 4). Their molar activities at EOS were 390 ± 230 GBq/µmol (*n* = 19) and 280 ± 130 GBq/µmol (*n* = 37), respectively. The total synthesis times were 76–89 min and 89–99 min after irradiation, respectively.

QC testing results for the three different batches of [^18^F]SPAL-T-06 and [^18^F]C05-05 are summarized in Table 4. All QC results for the two PET radiopharmaceuticals complied with in-house QC and quality assurance specifications (Table 1). These data (Table 4) demonstrate the reliable production of the two PET radiopharmaceuticals with sufficient radioactivity and high quality to support clinical investigations.

**Table 4:** Results of quality control testing of the [^18^F]SPAL-T-06 and [^18^F]C05-05 injections

	[^18^F]SPAL-T-06			[^18^F]C05-05		
Specification	Lot 1	Lot 2	Lot 3	Lot 1	Lot 2	Lot 3
Volume (mL)	16.7	16.4	16.5	15.1	16.3	16.5
Radioactivity (GBq)	1.03	1.16	0.90	1.42	1.86	2.65
Molar activity (GBq/µmol)	407	959	406	242	334	231
Half-life (min)	107	111	112	113	106	110
Appearance	Pass^a^	Pass^a^	Pass^a^	Pass^a^	Pass^a^	Pass^a^
Bacterial endotoxin	Pass^b^	Pass^b^	Pass^b^	Pass^b^	Pass^b^	Pass^b^
Sterility	Pass^c^	Pass^c^	Pass^c^	Pass^c^	Pass^c^	Pass^c^
pH	5.5	5.7	5.8	5.8	5.8	5.6
Radionuclidic identity	Pass	Pass	Pass	Pass	Pass	Pass
Radiochemical identity (%)	1.2	1.4	0.9	1.7	1.2	2.0
Radionuclidic purity (%)	≥ 99.9	≥ 99.9	≥ 99.9	≥ 99.9	≥ 99.9	≥ 99.9
Radiochemical purity at EOS* (%) Radiochemical purity at 180 min after EOS* (%)	99.9 99.9	99.1 99.9	99.1 99.7	99.9 99.9	99.9 99.9	99.9 99.9
Kryptofix 222 (µg/mL)	< 40	< 40	< 40	< 40	< 40	< 40
Residual solvents	Pass	Pass	Pass	Pass	Pass	Pass
Ethanol (µg/mL) Acetonitrile (µg/mL) Dimethyl sulfoxide (µg/mL)	2.9 0.2 < 0.1	2.8 0.3 < 0.1	3.1 0.2 < 0.1	2.1 0.2 < 0.1	1.6 0.3 0.7	1.7 0.3 0.1

^*^EOS: End of synthesis. ^a^Clear colorless liquid with no suspended particles. ^b^ < 1.10 international units per maximum dose. ^c^No bacterial growth

### Stabilities of [^18^F]SPAL-T-06 and [^18^F]C05-05 under standard fluorescent light or UV-cut off light

**Fig. 5:**
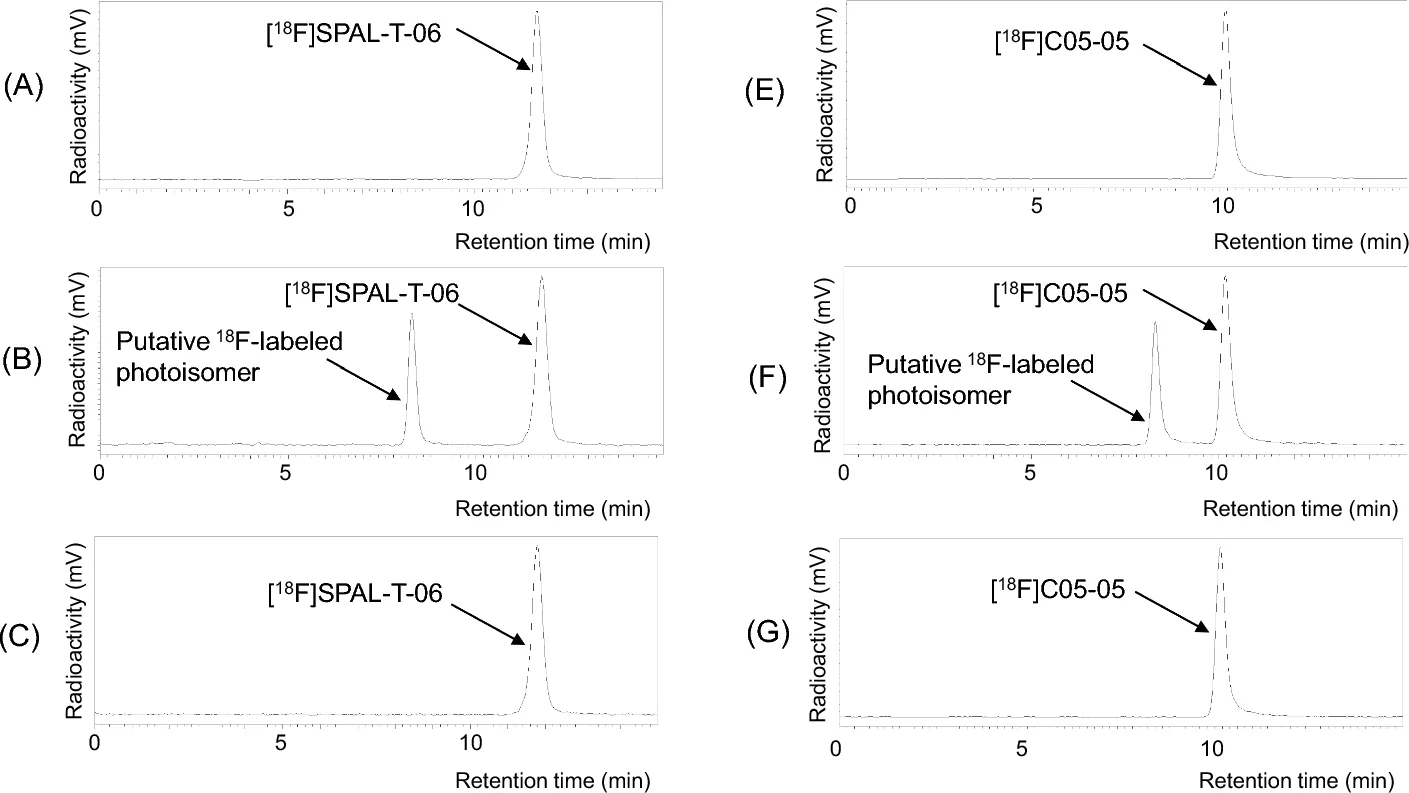
Typical analytical HPLC chromatograms of [^18^F]SPAL-T-06 (**A**, **B**, and **C**) and [^18^F]C05-05 (**D**, **E**, and **F**) obtained using radio-HPLC. HPLC analyses were performed immediately after formulation (**A** and **D**), 30 min after exposure to standard fluorescent light (**B** and **E**), and 60 min after exposure to UV-cutoff light in a natural light-shielded environment (**C** and **F**). The analytical HPLC conditions were as follows: an ACE Excel 3 C18-Amide column (3 μm, 4.6 mm i.d. × 100 mm), a mixture of 90% acetonitrile and 100 mmol/L ammonium phosphate buffer (pH 2.0) containing 5 mmol/L sodium 1-octanesulfonate (42:58 [v/v] for [^18^F]SPAL-T-06 or 35:65 [v/v] for [^18^F]C05-05) as the mobile phase, a flow rate of 1.0 mL/min, UV detection at 390 nm, and an injection volume of 5 μL

**Fig. 6:**
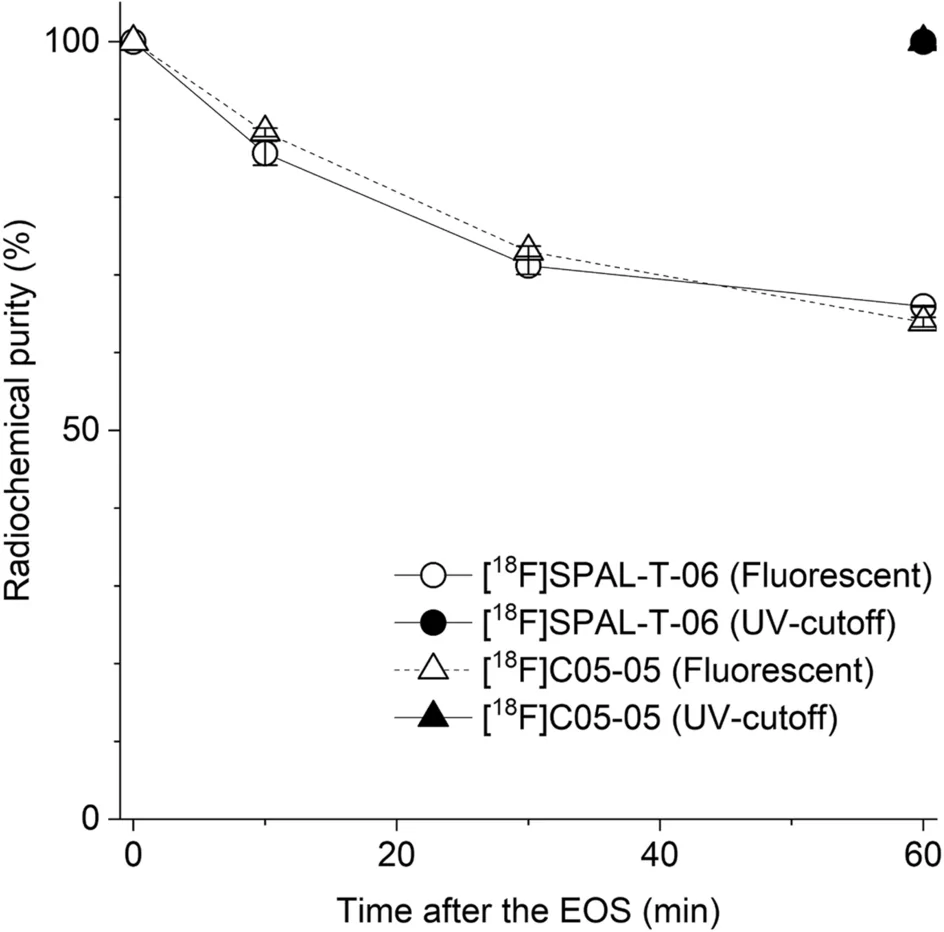
Radiochemical purities of [^18^F]SPAL-T-06 and [^18^F]C05-05 after exposure to standard fluorescent light in a general laboratory environment and to UV-cutoff light in a natural light-shielded environment. Radiochemical purities at each time point were measured in triplicate

In a natural light-shielded environment, the radiochemical purities of [^18^F]SPAL-T-06 and [^18^F]C05-05 exceeded 99% after 60 min (Fig. 5). In addition, under UV-cutoff light in a natural light-shielded environment, the radiochemical purities of [^18^F]SPAL-T-06 and [^18^F]C05-05 exceeded 99% after 60 min (Figs. 5 and 6). After 10 min of exposure to standard fluorescent light in a general laboratory environment, the radiochemical purities of [^18^F]SPAL-T-06 and [^18^F]C05-05 decreased by approximately 86% (Fig. 6). After 30 min of exposure to standard fluorescent light in a general laboratory environment, the radiochemical purities of [^18^F]SPAL-T-06 and [^18^F]C05-05 continued to decrease, reaching approximately 64% at 60 min (Fig. 6). In contrast, the radiochemical purities of [^18^F]SPAL-T-06 and [^18^F]C05-05 remained above 99% after 60 min under UV-cutoff light (Fig. 6). Furthermore, the radiochemical purities of each radiopharmaceutical exceeded 99% after 180 min in a natural light-shielded environment without fluorescent or UV-cutoff light.

## Discussion

We successfully prepared two PET radiopharmaceuticals, [^18^F]SPAL-T-06 and [^18^F]C05-05, for clinical investigation using an ^18^F-labeling synthesizer. In the radiosynthesis of [^18^F]PM-PBB3 which is a leading tracer for [^18^F]SPAL-T-06 and [^18^F]C05-05, we previously optimized ^18^F-fluorination reaction condition using a lower concentration of K_2_CO_3_ solution (Kawamura et al. 2021). Herein, the radiosyntheses of [^18^F]SPAL-T-06 and [^18^F]C05-05 were performed using ^18^F-fluorination under the same reaction conditions as the radiosynthesis of [^18^F]PM-PBB3. By examining the deprotection reagents and conditions for [^18^F]SPAL-T-06, the RCY (n.d.c) at EOS improved from 4.7 to 11% (Tables 2 and 3); however, the RCY (n.d.c) of [^18^F]SPAL-T-06 at EOS was lower than that of [^18^F]PM-PBB3 (25%; Table 3; Kawamura et al. 2021). Similarly, the RCY (n.d.c) of [^18^F]C05-05 at EOS (20%; Table 3) was lower than that of [^18^F]PM-PBB3 (25%; Table 3). The efficiency of ^18^F-fluorination may be attributed to structural variations between the carbon–carbon (C–C) triple and double bonds, as [^18^F]SPAL-T-06 and [^18^F]C05-05 each contain one C–C triple bond, whereas [^18^F]PM-PBB3 contains two C–C double bonds. In general, a C–C triple bond is more electron deficient than a C–C double bond and is more susceptible to hydration under acidic conditions. Therefore, the presence of a C–C triple bond in [^18^F]SPAL-T-06 and [^18^F]C05-05 may interfere with ^18^F-fluorination efficiency more readily than the C–C double bonds in [^18^F]PM-PBB3. Furthermore, [^18^F]SPAL-T-06 showed a lower RCY (n.d.c) than [^18^F]C05-05 (Table 3), which was likely due to the structural differences between the pyrazine ring in [^18^F]SPAL-T-06 and the pyridine ring in [^18^F]C05-05. Potential effects of these structural differences on RCY (n.d.c) will be investigated in future radiosyntheses of more derivatives containing pyridine and pyrazine moieties in this series of compounds.

In our previous studies, [^11^C]PBB3 and [^18^F]PM-PBB3 underwent rapid photoisomerization to form pyridylbutadienylbenzothiazole compounds upon exposure to fluorescent light (Hashimoto et al. 2014; Kawamura et al. 2021). After exposure to standard fluorescent light for 5 min, the radiochemical purity of [^18^F]PM-PBB3 was approximately 24% (Kawamura et al. 2021). In the present study, the radiochemical purities of [^18^F]SPAL-T-06 and [^18^F]C05-05 exceeded 86% after 10 min of exposure to standard fluorescent light. This difference may be explained by the fact that [^18^F]SPAL-T-06 and [^18^F]C05-05 contain one C–C double bond, whereas [^18^F]PM-PBB3 contains two. Therefore, [^18^F]SPAL-T-06 and [^18^F]C05-05 were more stable under standard fluorescent light than [^18^F]PM-PBB3. Furthermore, the radiochemical purities of [^18^F]SPAL-T-06 and [^18^F]C05-05 exceeded 99% after 60 min in a natural light-shielded environment under UV-cutoff light only. Thus, photoisomerization induced by exposing [^18^F]SPAL-T-06 and [^18^F]C05-05 to standard fluorescent light was effectively prevented by using UV-cutoff light in a natural light-shielded environment.

## Conclusions

We prepared [^18^F]SPAL-T-06 and [^18^F]C05-05 as PET radiopharmaceuticals for clinical investigations. The preparation and QC procedures established in this study provide a practical basis for standardizing protocols and technology transfer to other PET centers. In particularly, all post-deprotection radiosynthesis steps and QC testing for [^18^F]SPAL-T-06 and [^18^F]C05-05 should be performed under UV-cutoff light in a natural light-shielded environment to prevent photoisomerization.

## Supplementary Information

Additional file1 (DOCX 239 KB)

## Data Availability

The data supporting the findings of this study are provided within the article.

## Abbreviations

PET: Positron emission tomography
QC: Quality control
EOS: End of synthesis
HPLC: High-performance liquid chromatography
GC: Gas chromatography
THP: Tetrahydropyranyl
DMSO: Dimethyl sulfoxide
TFA: Trifluoroacetic acid
RCY (n.d.c): Non-decay-corrected radiochemical yield
