# Supplementary material for: Radiosynthesis and quality control testing of two 18F-labeled radiopharmaceuticals for α-synuclein imaging in clinical investigations
Source: EJNMMI Radiopharm Chem. 2026 Jul 23;11:76. doi: 10.1186/s41181-026-00480-w (PMC13677666; doi:10.1186/s41181-026-00480-w)
Supplement: Supplementary file 1 [file 41181_2026_480_MOESM1_ESM.docx]

**Additional file of “Radiosynthesis and quality control testing of two ^18^F-labeled radiopharmaceuticals for α-synuclein imaging in clinical investigations”**

**Supplementary materials**

**Fig. S1**

Fig. S1. Calibration curves of SPAL-T-06 (A) and C05-05 (B) obtained using an analytical HPLC system (Waters). The analytical HPLC conditions were as follows: an ACE Excel 3 C18-Amide column (3 μm, 4.6 mm i.d. × 100 mm; Advanced Chromatography Technologies); a mixture of 90% acetonitrile and 100 mmol/L ammonium phosphate buffer (pH 2.0) containing 5 mmol/L sodium 1-octanesulfonate (42:58 [v/v] for [^18^F]SPAL-T-06 or 35:65 [v/v] for [^18^F]C05-05) as the mobile phase; a flow rate of 1.0 mL/min; UV detection at 390 nm; and an injection volume 5 μL. The reference standard solutions of SPAL-T-06 and C05-05 were measured in triplicates at concentrations of 0.01, 0.1, 1, and 10 μg/mL.

**Fig. S2.**

Fig. S2. Calibration curves of ethanol (A), acetonitrile (B) and DMSO obtained using a GC system (7890 Series, Agilent). The GC conditions were as follows: an injection volume 0.5 μL; a DB-WAX column (0.50 μm, 0.53 mm i.d. × 30 m; Agilent Technologies); the column temperature was initially set at 40 °C (0–8 min), then increased to 120 °C (8–28 min), and finally maintained at 120 °C (28–33 min); nitrogen (99.99995%, 50 mL/min) was used as the makeup gas; helium (99.99995%, 4 mL/min) was used as the carrier gas; and detection was performed using a flame ionization detector (FID) with hydrogen (99.9999%) and compressed air as the combustion gases. The reference standard solutions of ethanol, acetonitrile, and DMSO were measured in triplicate at concentrations of 10, 100, and 1,000 μg/mL.

**Fig. S3**

Fig. S3. Typical semi-preparative HPLC chromatograms of [^18^F]SPAL-T-06 (A) and [^18^F]C05-05 (B) obtained using a radio-HPLC system. The HPLC conditions were as follows: an Inertsil ODS-HL column (5 μm, 10 mm i.d. × 250 mm length, GL Science); a mixture of acetonitrile and 50 mmol/L ammonium acetate solution (44:56 [v/v] from 0 to 48 min and then 100% acetonitrile for [^18^F]SPAL-T-06 or 50:50 [v/v] from 0 to 47 min and then 100% acetonitrile for [^18^F]C05-05) as the mobile phase; a flow rate of 5.0 mL/min; and UV detection at 390 nm.
